# Estimating relative biomasses of organisms in microbiota using “phylopeptidomics”

**DOI:** 10.1186/s40168-020-00797-x

**Published:** 2020-03-06

**Authors:** Olivier Pible, François Allain, Virginie Jouffret, Karen Culotta, Guylaine Miotello, Jean Armengaud

**Affiliations:** 1 Laboratoire Innovations technologiques pour la Détection et le Diagnostic (Li2D), Service de Pharmacologie et Immunoanalyse (SPI), CEA, INRAE, F-30207 Bagnols-sur-Cèze, France; 2 Laboratory “Innovative technologies for Detection and Diagnostics”, DRF-Li2D, CEA-Marcoule, BP 17171, F-30200 Bagnols-sur-Cèze, France

**Keywords:** Biomass, Metaproteomics, Microbiome, Shared peptides, Signature, Tandem mass spectrometry

## Abstract

**Background:**

There is an important need for the development of fast and robust methods to quantify the diversity and temporal dynamics of microbial communities in complex environmental samples. Because tandem mass spectrometry allows rapid inspection of protein content, metaproteomics is increasingly used for the phenotypic analysis of microbiota across many fields, including biotechnology, environmental ecology, and medicine.

**Results:**

Here, we present a new method for identifying the biomass contribution of any given organism based on a signature describing the number of peptide sequences shared with all other organisms, calculated by mathematical modeling and phylogenetic relationships. This so-called “phylopeptidomics” principle allows for the calculation of the relative ratios of peptide-specified taxa by the linear combination of such signatures applied to an experimental metaproteomic dataset. We illustrate its efficiency using artificial mixtures of two closely related pathogens of clinical interest, and with more complex microbiota models.

**Conclusions:**

This approach paves the way to a new vision of taxonomic changes and accurate label-free quantitative metaproteomics for fine-tuned functional characterization.

Video abstract

## Background

Understanding the functioning of microbial consortia and more sophisticated ecosystems through the analysis of their structure and biological interactions is important in many fields, including medicine and environmental ecology. Metaproteomics has recently emerged as a powerful analytical tool for studying the protein content of complex environmental and medical samples [1–4]. Combined with rapidly improving methods for sample preparation, mass spectrometry acquisition, and data interpretation [5, 6], metaproteomics has the potential to have a major impact on ecosystem analysis by establishing the diversity of microorganisms present in biological samples, estimating their biomass, and documenting the central metabolic pathways in each organism. Numerous applications are expected in the near future, but more robust per-species and per-function quantitation data are required [7].

Several generic approaches based on high-throughput proteomics have been proposed for identifying organisms without a priori knowledge [8–10]. Mixed species samples can be analyzed by shotgun proteomics, their content deciphered in terms of taxonomy, and their relative abundances roughly estimated, as shown with mixtures of bacteria [10, 11]. The taxonomic level at which each peptide is discriminative can be established with tools such as Unipept, based on a search of the lowest common ancestor [12]. Assessment of peptidome similarity is also a potentially powerful technique for peptide-centric representation of microorganisms and their identification [13]. Such approaches are gaining ground, especially with the development of metaproteomics pipelines [6, 14–16].

Identification and quantification methods relying on discriminative peptides have several limitations. Ideally, the protein databases used should be constructed only with fully sequenced bacterial genomes rigorously filtered and curated [17]. If a database is overpopulated, to give a more comprehensive view of the microbial diversity, then its size dramatically increases, making its search fastidious but also decreasing the number of specific peptides, as previously demonstrated [18, 19]. Accordingly, database composition has a strong influence on protein inferences and additional genomes may lead to cross-species protein matches and misidentification of microorganisms. Furthermore, when metaproteomics is performed on complex samples containing a wide diversity of organisms, many species-specific peptides will be present in lower abundance compared with peptides shared between taxa, which will be more abundant. Because of their higher abundance, the latter are systematically favored and selected for fragmentation by tandem mass spectrometry in “shotgun” experiments. Consequently, the relative quantification of microorganisms may be erroneous if based only on a limited set of discriminative species-specific peptides.

To solve the “less discriminative peptides if the database is more comprehensive” paradox and improve the relative quantification of microorganisms by metaproteomics, we propose to consider peptides shared between taxa. While being systematically rejected or outlined as a skew factor in hitherto metaproteomics methodologies, they contain information to improve discrimination and quantification of taxa. To take advantage of their value, a phylogenetic distance can be systematically computed for almost (86%) all sequenced organisms, which allows the use of the full peptidome to analyze any organism mixture. Here, we describe a mathematically computed signature profile predicting the amount of shared peptides in all organisms due to a given organism, usable to depict the full tandem mass spectrometry signal in terms of a linear combination of such organism signatures. We term our approach “phylopeptidomics” as it stems on phylogenetic and peptidome data at omics level. We illustrate the capacity of phylopeptidomics to estimate with precision the relative abundances of organisms used in artificial mixtures of two closely related pathogens, *Salmonella bongori* and *Shigella flexneri*, and its applicability for more complex microbiota models.

## Results

### Shared peptides are informative: from taxonomical proximity to phylogenetic distance dependency

**Fig. 1:**
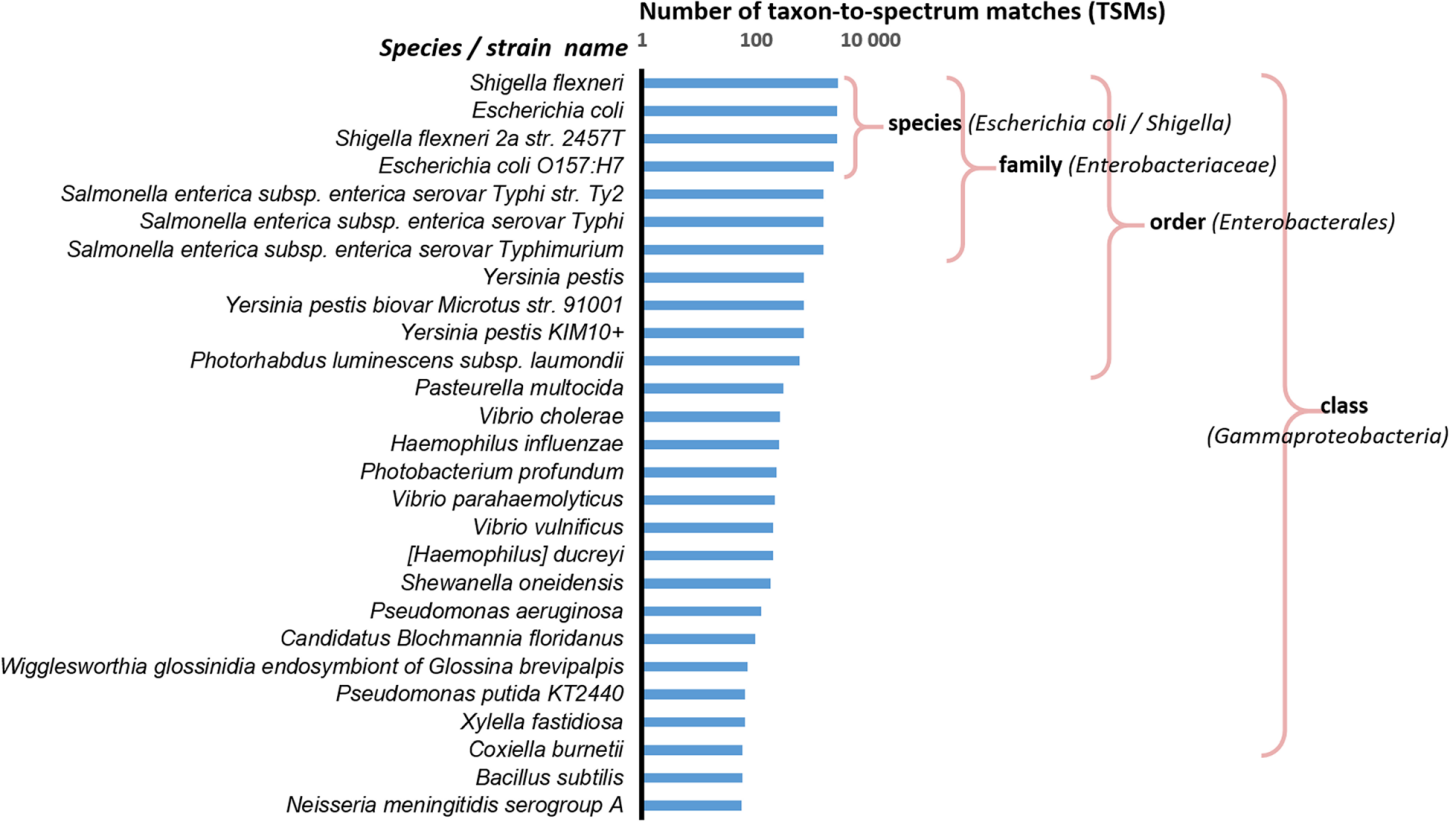
Number of taxon-to-spectrum matches for a pure *Shigella flexneri* sample. MS/MS spectra that can be associated with peptides and proteins to a selection of taxa available from Ciccarelli et al. [20] were numbered. A key observation is that the number of taxon-to-spectrum matches attributed to taxa that are not in the sample is directly linked to the taxonomical proximity to *Shigella flexneri*

We assigned the MS/MS spectra recorded on the *S*. *flexneri* proteome against the comprehensive National Center for Biotechnology Information non-redundant (NCBInr) database, the largest repertoire of genome-derived protein sequences. We examined the number of peptide-to-spectrum matches (PSMs) assigned to different taxa without the application of parsimony analysis, such that each MS/MS spectrum can be assigned to several peptides, and each PSM can be assigned to various taxa (Fig. 1). We propose to name those MS/MS spectra assigned to taxa as taxon-to-spectrum matches (TSMs). We ranked these results from the highest to the lowest number of TSMs (Fig. 1). Because *S*. *flexneri* contains a large fraction of sequences similar to other *Shigella* and *Escherichia* species, these two genera share a large number of TSMs. For the pure *S*. *flexneri* sample, a clear relationship was found between the number of TSMs between organisms and their taxonomical relationships. Clearly, the distribution of TSMs (Fig. 1) will depend on the number of organisms present in the database, and new genome-sequenced organisms can be added to this distribution when they become available. The chart conveys the idea that the number of shared PSMs, and thus TSMs, is correlated with the phylogenetic distance of the organism present in the sample to those present in the database and can be theoretically estimated. To model the abundance of shared peptides between an organism present in a sample and any other taxa, we propose to calculate their phylogenetic distances and display the results (Fig. 1) along these distances.

**Fig. 2:**
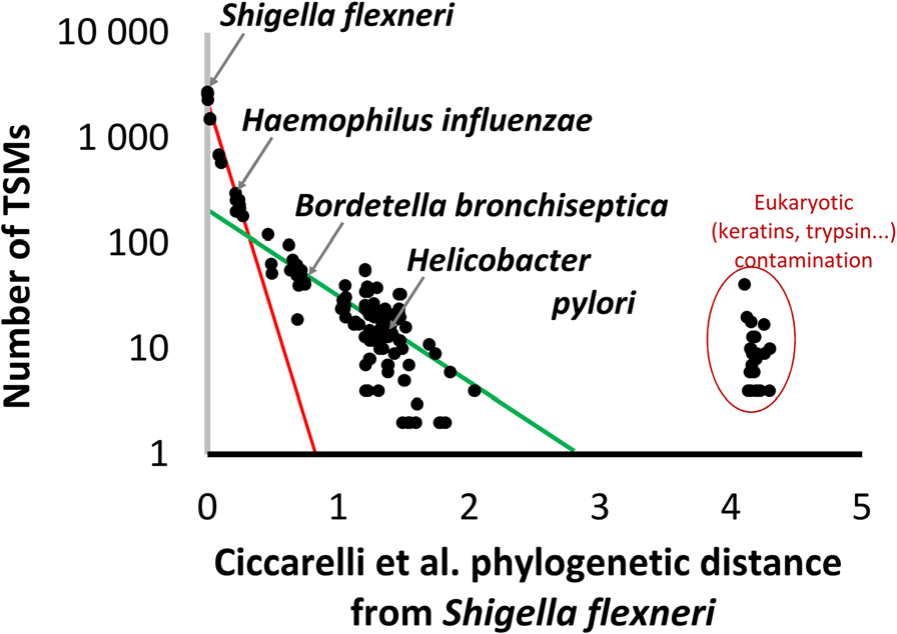
Schematized correlation of the number of taxon-to-spectrum matches with the phylogenetic distance from *Shigella flexneri*, for a pure *S*. *flexneri* sample. Distances associated with taxa as in Fig. 1 and calculated from Ciccarelli et al. [20] are reported on the *x*-axis in logarithmic scale. The number of TSMs associated with taxa are reported on the *y*-axis in logarithmic scale. We noted a possible correlation between the number of TSMs and the distance. Two possible exponential terms for this correlation are schematized in red and green. Some eukaryotic taxa show an additional signal in relation to proteins actually in the sample (keratins, trypsin…)

To analyze the possible relationship, we plotted taxa along the horizontal axis with values corresponding to the phylogenetic distances from taxon *S*. *flexneri* estimated with a matrix of distances for 191 organisms based on clusters of orthologous groups of proteins (COGs) that are ubiquitous across superkingdoms [20], and the values corresponding to the number of TSMs for each taxon along the vertical axis (Fig. 2). With the vertical axis in logarithmic scale, a bilinear function (lines in red and in green) could be clearly observed for the microorganisms, meaning that the curve can be modeled by the sum of two exponential terms. Additional signals are seen at the highest distance from *S*. *flexneri*, corresponding to eukaryotic-specific signals (mammal keratins, trypsin) that can be pre-filtered against a contaminant database. This representation supports the idea that it is possible to model the abundance of shared peptides (here evaluated by their spectral counts) between an organism present in a sample and any other taxa, based solely on their phylogenetic distance, using the fit of functions—which we have termed phylopeptidomic signatures—to the experimental proteomics data.

### Automatic phylogenetic distance matrix computation for the three domains-of-life

To generate an overall phylogenetic distance matrix for eukaryotes, archaea, and bacteria, we curated the multiple sequence alignment (MSA) initially proposed by Ciccarelli et al. for 180 organisms [20], and automated a process for incorporating the tens of thousands of sequenced organisms. A global MSA of supervectors of all concatenated masked COGs was used to generate a complete percent identity matrix (PIM) for proteomes of 14,237 taxa. The resulting multiple alignment considering 8310 amino acid positions is applicable to inter-domain comparison. Its performance for estimating phylogenetic distances compared favorably with 16S rRNA calculated distances for a selection of *Shigella*, *Escherichia*, and *Salmonella* strains (Supplementary Figure S1). Remarkably, the COG *x*-axis allowed a much clearer separation of pairs of taxa, in full accordance with the exact taxonomical classification of the species, suggesting an improvement in precision and accuracy for this pan-domains-of-life MSA.

### Validation of the phylopeptidomic signature concept with experimental MS/MS datasets

**Fig. 3:**
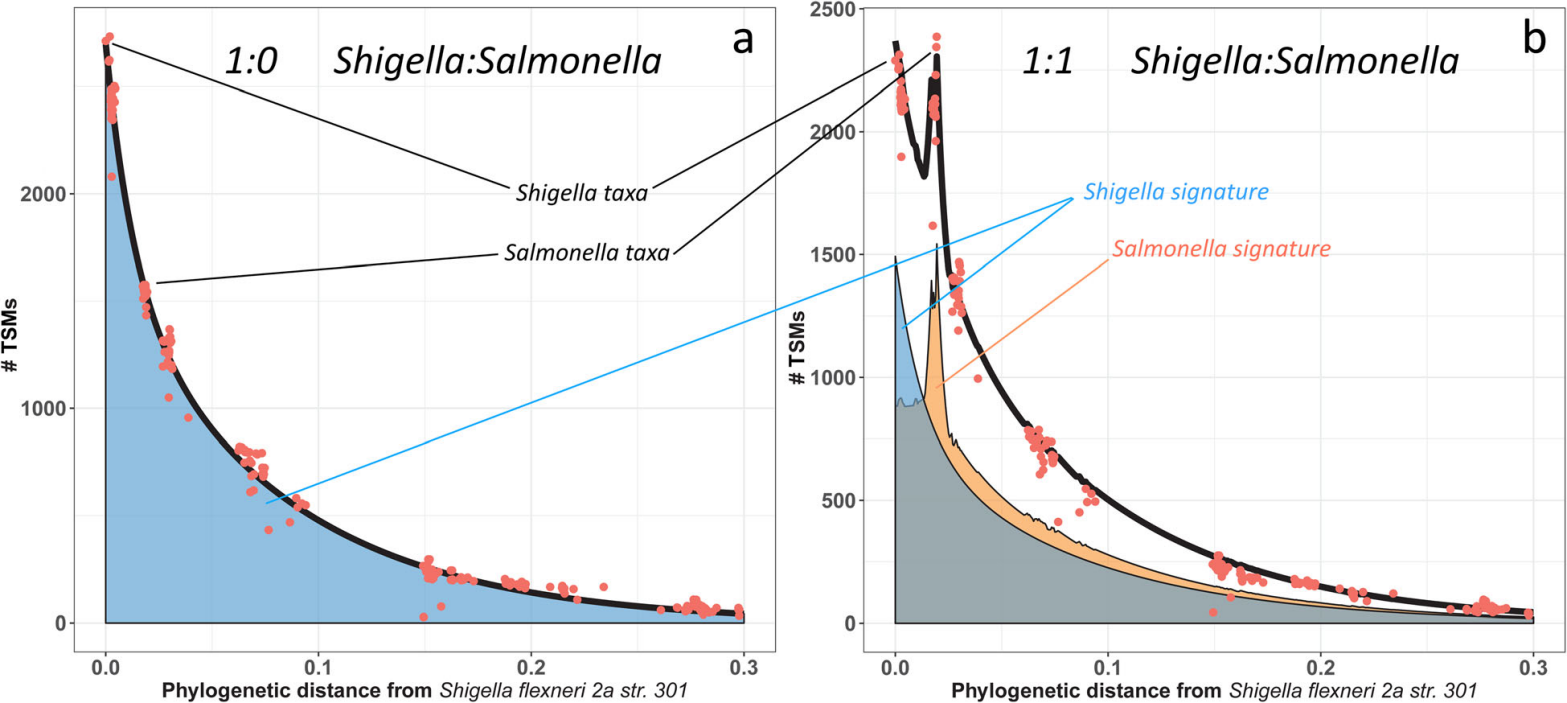
Fit of phylopeptidomics signatures for two experimental proteomic datasets. MS/MS datasets were acquired on a pure *Shigella flexneri* sample (**a**), and a 1:1 *S*. *flexneri*:*Salmonella bongori* mixture (**b**). Instead of the phylogenetic distances for 191 organisms as available in Ciccarelli et al. [20], the distances used here are calculated as detailed in the “Methods” section for 14,237 taxa. The phylogenetic distance from taxon *S*. *flexneri 2a str*. *301* is plotted on the *x*-axis and is calculated as detailed in the “Results” section. The phylopeptidomics signature of a single organism REF is:${y}_{i, REF}={N}_{REF1}\times \left({A}_{REF}\times \kern0.45em {e}^{-\frac{x_{i, REF}}{a_{REF}}}+\left(1-{A}_{REF}\right)\times \kern0.33em {e}^{-^{-\frac{x_{i, REF}}{b_{REF}}}}\right)$with *i* any taxon, *y*_*i*, REF_ the number of TSMs of taxon *i* due to organism REF, *x*_*i*, REF_ the phylogenetic distance between *i* and REF, *a*_REF_ and *b*_REF_ coefficients for the two exponential terms, *A*_REF_ a repartition term, and *N*_REF_ the term indicating the quantity of organism REF. **a** Sample L00221 from Supplementary Table SI, corresponding to a 1:0 *Shigella*:*Salmonella* ratio. The display is along *x*_*i*, REF1_*x*-axis with REF1 = *S*. *flexneri 2a str*. *301*, REF2 = *S*. *bongori NCTC 12419*, *N*_REF1_ = 2697 and *N*_*REF*2_ = 21 as fitted. The *S*. *flexneri 2a str*. *301* signature is displayed in a blue filled area. **b** Sample L00306 from Supplementary Table SI, corresponding to a 1:1 *Shigella*:*Salmonella* ratio. The display is along *x*_*i*, REF1_*x*-axis with REF1 = *S*. *flexneri 2a str*. *301*, REF2 = *S*. *bongori NCTC 12419*, *N*_REF1_ = 1492, and *N*_REF2_ = 1558 as fitted. The *S*. *flexneri 2a str*. *301* and *S. bongori NCTC 12419* signatures are displayed respectively in blue- and orange-filled areas. The relative percentage of *S*. *flexneri* calculated using phylopeptidomics signatures is thus 1492/3050 = 49% compared with 50% expected

Three biological replicates of the *S*. *flexneri* proteome were analyzed by shotgun tandem spectrometry along a gradient of 90 min. The resulting dataset comprised 9827, 10,518, and 9540 MS/MS spectra that were tested against the NCBInr database, resulting in 2794, 3887, and 3344 PSMs, respectively (Supplementary Table SI). As expected from such an inflated database, the ratio of PSMs compared with MS/MS spectra was rather low (33.5%), but the dataset is sufficiently informative. The number of unique peptide sequences was 1746, 2512, and 2088, respectively. TSMs were extracted and, as expected, the highest value was obtained for *S*. *flexneri*, with 2753, 3806, and 3283 TSMs for the three biological replicates. TSM values for all the taxa listed in the NCBInr database were plotted along their phylogenetic distances from *S*. *flexneri* for replicate L00221 (Fig. 3a). The signature of *S*. *flexneri* for describing the ratio of shared peptides with any other taxon was modeled using the function:

$$ {y}_{i, REF}={N}_{REF}\times \left({A}_{REF}\times \kern0.45em {e}^{-\frac{x_{i, REF}}{a_{REF}}}+\left(1-{A}_{REF}\right)\times \kern0.33em {e}^{-^{-\frac{x_{i, REF}}{b_{REF}}}}\right) $$

where REF is the taxon of which the function is the signature (here, *S*. *flexneri*), *i* any taxon, *y*_*i*, REF_ is the number of TSMs of taxon *i* due to taxon REF at quantity *N*_REF_, *N*_REF_ is the number of TSMs assigned to taxon REF and indicates the quantity of MS/MS-detected REF, *A*_REF_ is a coefficient to adjust the proportions of each exponential term, *x*_*i*, REF_ is the phylogenetic distance between taxon i and taxon REF, *a*_REF_ is the first decreasing exponential coefficient, and *b*_REF_ is the second decreasing exponential coefficient. Proportions of each exponential term depend upon the depth of mass spectrometry analysis of each experiment and must therefore be adjustable, using the *A*_REF_ coefficient. For the three *S*. *flexneri* MS/MS datasets, calculated coefficients *a*_REF_ and *b*_REF_ were similar, 0.013 and 0.082, respectively, and the *A*_REF_ coefficients were 0.4, 0.44, and 0.45 (Supplementary Table SI). For each specific dataset involving *S*. *flexneri*, the phylopeptidomic signature can be calculated and used to predict the number of TSMs of any taxon because of the shared peptides between *S*. *flexneri* and this taxon.

### Acquisition of experimental datasets on laboratory-assembled mixtures of two closely related bacteria and their accumulated phylopeptidomic signature

We analyzed several laboratory-assembled mixtures of *S*. *flexneri* and *S*. *bongori* (1:0, 1:0.1, 1:0.2, 1:0.5, 1:1, 0.5:1, 0.2:1, 0.1:1, and 0:1) in biological triplicates. Both species belong to the *Enterobacteriaceae* family and are animal pathogens. Because of their close phylogenetic distance, these Gram-negative bacteria are difficult to distinguish, and their relative quantitation represents a true challenge, as pathogen clades tend to be densely sequenced due to their clinical interest. The peptide sequences identified against the NCBInr database were associated with all the taxa listed in this database. The number of MS/MS spectra recorded and TSMs per taxa assigned for the 27 samples at different taxonomical levels (superkingdom, phylum, class, order, family, genus, species, and strain) are listed in Supplementary Table SI. We also listed the number of specific peptide sequences and their spectral counts at all taxonomical levels. A clear outcome of the spectra to taxa inference was that the higher the taxonomical level considered, the more numerous were the specific peptides and specific TSMs. For example, for the 1:1 ratio of *S*. *flexneri* and *S*. *bongori*, the average ratio of specific TSMs compared with the total number of PSMs was 47% at the phylum level, ranged from 34% to 37% between class and family levels, and dropped to 12% for *Salmonella* and less than 1% for *Shigella* at the genus level.

Considering the presence of *S*. *flexneri* 2a str. 301 (REF1) and *S*. *Bongori* NCTC 12419 (REF2) as the organisms present in the laboratory-assembled mixtures, their global TSM signal was modeled as the sum of two phylopeptidomic signatures with the following function:

$$ {y}_{i, REF1}+{Y}_{i, REF2}={N}_{REF1}\times \left({A}_{REF1}\times \kern0.45em {e}^{-\frac{x_{i, REF1}}{a_{REF1}}}+\left(1-{A}_{REF1}\right)\times \kern0.33em {e}^{-^{-\frac{x_{i, REF1}}{b_{REF1}}}}\right)+{N}_{REF2}\times \left({A}_{REF2}\times \kern0.45em {e}^{-\frac{x_{i, REF2}}{a_{REF2}}}+\left(1-{A}_{REF2}\right)\times \kern0.33em {e}^{-^{-\frac{x_{i, REF2}}{b_{REF2}}}}\right) $$

This function gives the number of TSMs of any other taxa due to their shared peptides with the two organisms present in the sample (REF1 and REF2) and detected at different levels considering their abundance in the sample (*N*_REF1_ and *N*_REF2_). The parameters of this function were calculated for each experimental dataset to minimize the quadratic sum of errors between the experimental *y*_*i*_ number of TSMs for taxon i and the sum of adjusted *y*_*i*, REF1_ and *y*_*i*, REF2_. The parameter adjustment reflecting directly REF1 and REF2 proteomic abundances was that of *N*_REF1_ and *N*_REF2_. Figure 3b shows the sum of both signatures automatically adjusted for the metaproteomics signal of 1:1 *S*. *flexneri*:*S*. *bongori* mixture from replicate L00273 using *x*_*i*, REF1_*x*-axis only. Similar displays could be plotted using *x*_*i*, REF2_*x*-axis. As shown, the phylopeptidomic global signature fitted perfectly with the experimental signals. Such representation highlights the presence of the two bacteria as discrete signals detected at their phylogenetic distance. For this mixture, the MS/MS spectra datasets were 9928, 9327, and 9426 for the three biological replicates (Supplementary Table SI). The numbers of PSMs assigned to the NCBInr database were 3524, 3021, and 3174. The numbers of TSMs assigned to *S*. *flexneri* 2a str. 301 were 2464, 2157, and 2297, whereas those assigned to *S*. *bongori* NCTC12419 were slightly more numerous at 2716, 2363, and 2385. The mean percentage obtained by phylopeptidomic deconvolution of the three replicates for the *S*. *shigella* signal was 47% (± 1%), which is in good agreement with the theoretical abundance value for this mixture.

### Phylopeptidomic relative quantification gives a linear response

**Fig. 4:**
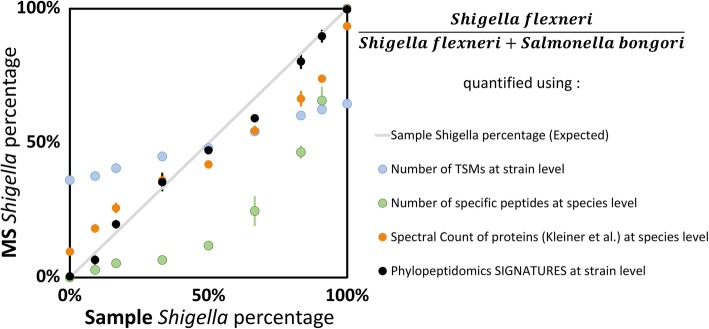
*Shigella* ratios deduced from the contributions of *S*. *flexneri 2a str*. *301* and *Salmonella bongori NCTC 12419* strain signatures. The *Shigella* ratios (black circles) are in very good agreement with sample experimental ratios (linear regression *R*^2^, 0.993; slope, 0.97; *R*^2^ to expected, 0.992). Ratios calculated from specific peptides (medium blue circles) that (i) can only be calculated at species level and (ii) are biased by the high sequencing density and the phylogenetic proximity of the *Shigella*/*Escherichia* genera, reducing the number of specific peptides for *Shigella flexneri* and underestimating *Shigella flexneri* percentages (*R*^2^ to expected, 0.647). Ratios estimated from the number of peptide-to-spectrum matches for the two strains (light blue circles) are highly biased by the high amount of shared peptides between both organisms (*R*^2^ to expected, 0.666). Ratios estimated using the sum of spectral counts of proteins with more than two unique peptides (orange circles), as proposed by Kleiner et al. [11], are still biased by the shared peptides fraction (*R*^2^ to expected, 0.911). Error bars are ± 1SD based on biological replicates

The relative quantification results obtained by phylopeptidomic deconvolution for *S*. *flexneri* and *S*. *bongori* samples and the 7 laboratory-assembled mixtures are shown in Supplementary Table SII. All the replicates corresponded to the same reference quantity of 1.5 × 10^8^ bacterial cells. The ratios of both type of organisms were estimated using three different criteria: (i) the number of total TSMs for each organism at the strain level; (ii) the number of specific peptides at the species level, since there was not a sufficient number of specific peptides at the strain level due to the genome sequencing density of these pathogens; and (iii) the *N*_REF_ quantifications obtained by means of the calculated phylopeptidomic signatures (Fig. 4). The ratio estimated on the basis of the total TSMs assigned to each of the organisms is strongly biased by the number of shared peptides between organisms, leading, for example, to an evaluation of the 1:0.1 *S*. *flexneri*:*S*. *bongori* ratio at 62% instead of 91%, and an evaluation of the 1:0 *S*. *flexneri*:*S*. *bongori* ratio at 65% instead of 100%. Quantification relying on specific peptides is also biased by the strong dependency of specific peptides on genome sequencing density and taxonomical proximity of organisms, leading, for example, to an evaluation of the 1:0.1 *S*. *flexneri*:*S*. *bongori* ratio at 66% instead of 91%. A calculation of the relative biomasses of *Shigella* and *Salmonella* similar to the (2 PUP + Fido) method from Kleiner et al. [11] was also performed, using Mascot at 5% *p* value instead of SEQUEST+Fido at 5% false-discovery rate and selecting only those proteins with 2 unique peptides in the same NCBInr database containing both strains. The complete set of proteins identified is shown in Supplementary Table SIII, which lists for each protein the complete list of taxa associated with proteins of identical sequence and proteins matching the same sets of peptides that are undistinguishable from the listed proteins. This approach gave results closer to the expected curve but was still sensitive mostly to the shared peptides fraction (Fig. 4). The mean absolute percentage errors were 36.1%, 30.2%, 12.7%, and 3.5% for the TSMs, specific peptides, Kleiner et al. [11], and phylopeptidomics methodologies, respectively. Phylopeptidomics-based quantification is both natively immune to the sequencing density and shared peptide effects and applicable to the most resolved taxonomical level. A linear response was obtained with a rather performant estimation of the ratio of bacteria. The coefficient of correlation (*R*^2^) at 0.9934 indicated a good linearity over the range of ratios tested. The standard deviation estimated with the three biological replicates was ~ 3%.

### Phylopeptidomics is applicable to complex samples

**Fig. 5:**
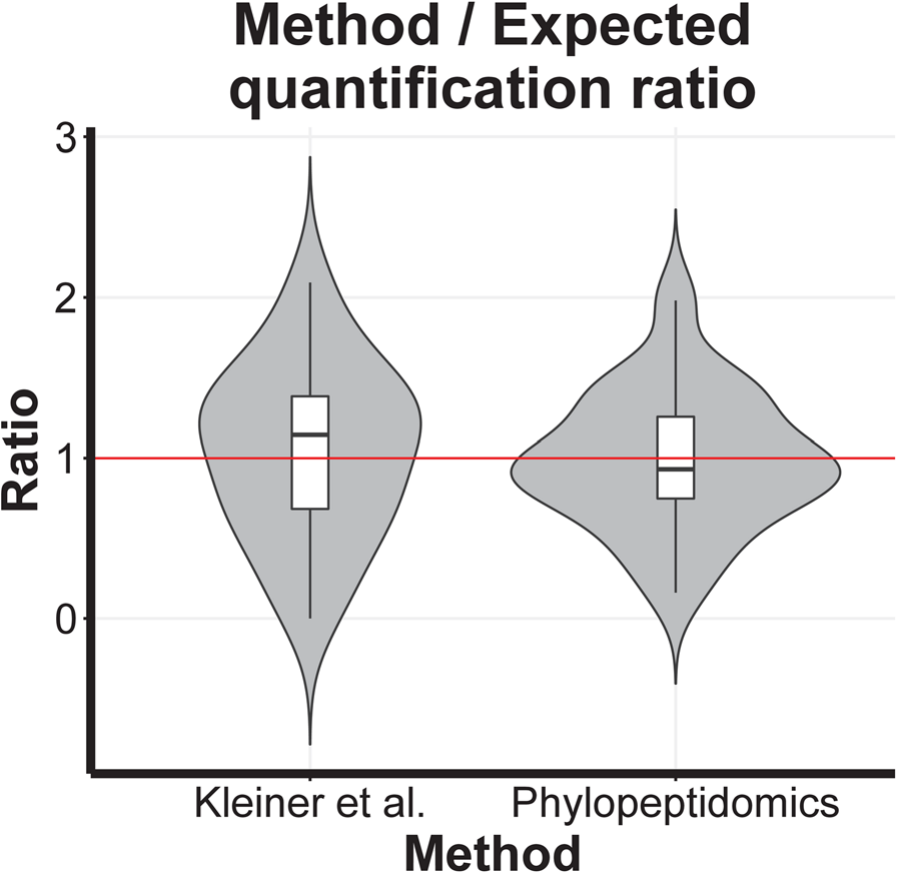
Violin plot of the ratios of the biomass percentage relative to the expected percentage for each species, for the method based on the protein quantification reported in Kleiner et al. and for the phylopeptidomics method. The sample used was Run5_P1_2000ng from Kleiner et al. [11], with an equal protein amount per organism. Results for strains from the same species have been cumulated into one species level result for *Staphylococcus aureus* and *Rhizobium leguminosarum*, virus results are not reported, and *Nitrosomonas europaea*, *Nitrosomonas ureae*, *Nitrosomonas multiformis*, and *Desulfovibrio vulgaris* are ignored as per Kleiner et al. [11] Supplementary Data 1, resulting in 19 species results compared as displayed in Supplementary Table SIV. The phylopeptidomics ratio median and distributions are closer to the target of 1, compared with that of Kleiner et al. [11] published results

A metaproteomic dataset of 39,363 MS/MS spectra was acquired on the ZymoBIOMICS Microbial Community Standard, which comprises five Gram-positive bacteria, three Gram-negative bacteria, and two fungi. The corresponding signatures for the ten microorganisms and the accurate fit to the overall proteomic signal plot are shown in Supplementary Figure S2. These results demonstrate the universal applicability of the phylopeptidomics signature. The dataset used for the biomasses quantitation includes 6163 taxa, with TSM values on the vertical axis, and 10 different phylogenetic distances displayed along the horizontal axis. This large dataset increases the statistical significance of the phylopeptidomics quantitation method and the resistance to outliers. The values obtained by (i) phylopeptidomics, as well as (ii) the expected biomasses calculated with an estimation of cell numbers and the mean volume of cells as proxy, are reported in Table 1. A relatively good correlation (*R*^2^ = 0.64) was obtained between ratios (i) and (ii) for the ten microorganisms. For example, *Lactobacillus fermentum* which is a rod-shaped bacterium 2 to 9 μm in length has a much larger volume than *Pseudomonas aeruginosa*. Thus, while their genomic content is similar, their protein content may strongly differ. This difference was clearly observed in the metaproteomics results with an estimated ratio of proteins of 24.2% and 1.9%, respectively. The exact cell quantities of each of the ten taxa in the ZymoBIOMICS Microbial Community Standard are not known, and the performances of phylopeptidomics cannot be precisely evaluated with this sample. Therefore, we applied phylopeptidomics to a larger MS/MS dataset of 143,804 MS/MS spectra acquired on a more complex sample comprising 22 bacterial species, 1 archaeal species, and 5 phage viruses in known quantities [11]. This large dataset was acquired with an extended chromatography run time adapted to the complexity of the sample. We extracted from Supplementary Data 1 [11] the protein amounts for each strain, in which the signal of four species, namely *Nitrosospira multiformis*, *Nitrosomonas europaea*, *Nitrosomonas ureae*, and *Desulfovibrio vulgaris*, was too weak to be quantified. The remaining 19 microorganism species could be quantified after calculation of their respective signatures and minimization of their combined signal (Supplementary Table SIV). We compared the quantitation results of phylopeptidomics and the Kleiner et al. [11] approach based on protein abundances (Fig. 5). Of note, a global quantitation ratio of 1.0 was obtained using the peptide-centric phylopeptidomics approach, whereas the protein-centric Kleiner et al. [11] approach tended to slightly overestimate this ratio. A paired bilateral *t* test with Supplementary Table SIV using “Kleiner/Expected ratio” column compared to a matching column filled with the expected values (1.0) gave a *p* value of 0.62, whereas the same statistic applied to the “Phylopeptidomics/Expected ratio” column gave a *p* value of 0.87, indicating a better match with phylopeptidomics. Furthermore, the results of phylopeptidomics were more uniform compared with the Kleiner et al. [11] method, as shown in the violin plot (Fig. 5).

**Table 1:** Relative quantification of organisms in the ZymoBIOMICS sample

Species	Genomic DNA composition (%)	Genome size (median total length in Mb)	Cell count ratio (with 1 genome copy)	Estimated cell volume (in μm^3^)	Relative biomass contributions—cell volume proxy	Signature quantitation TSMs^§^	Relative biomass contributions—phylopeptidomics signature at species level
*Pseudomonas aeruginosa*	12	6.58	6.50%	0.49	3.19%	271.3	1.9%
*Escherichia coli*	12	5.17	8.27%	0.65	5.37%	884.9	6.4%
*Salmonella enterica*	12	4.78	8.95%	0.96	8.61%	670.2	4.8%
*Lactobacillus fermentum*	12	1.99	21.49%	1.41	30.38%	3373	24.2%
*Enterococcus faecalis*	12	3.03	14.11%	0.52	7.39%	1002.8	7.2%
*Staphylococcus aureus*	12	2.85	15.01%	0.90	13.51%	2380.7	17.1%
*Listeria monocytogenes*	12	2.98	14.35%	0.27	3.85%	1163.2	8.4%
*Bacillus subtilis*	12	4.13	10.36%	2.69	27.90%	2946.1	21.2%
*Saccharomyces cerevisiae*	2	12.13	0.59%	33.51	19.69%	959.7	6.9%
*Cryptococcus neoformans*	2	19.05	0.37%	33.51	12.54%	267.9	1.9%

Cell counts were estimated based on one genome copy per cell. Cell volumes were roughly estimated based on the known morphology and dimensions of each species in classical growth conditions. The corresponding ratio of cell volumes is indicated as a possible proxy for biomass contributions assuming that the protein biomass may be correlated to the cell volume. These rough estimations are given only for an overview of possible biomass contributions for the ten species. The phylopeptidomic signature quantitation based on TSMs is indicated for each species as well as the corresponding calculated relative biomass contributions

^§^*TSM* taxon-to-spectrum matches

## Discussion

Both identification and estimation of biomass contribution are crucial objectives that should be achieved with the best sensitivity and precision to obtain the most comprehensive view of the structure and the temporal dynamics of a microbial community. Phylopeptidomics is a new approach that improves the estimation of biomass contributions by considering both discriminative peptides and peptides shared between organisms. It relies on a specific signature for a given organism, which describes the number and abundance of peptides shared with all other organisms depending on their phylogenetic distance. In this sense, the method we propose here is fundamentally different from the procedure presented by Kleiner et al. [11], which is protein-centered and not peptide-centered. Protein inference in metaproteomics is complex because of the large databases and the multiple occurrences of a large number of peptide sequences. In our approach, the higher redundancy introduced into the database, through the incorporation of additional new genome sequences, does not increase possible biases but rather benefits directly for a better fit of the signatures of shared peptides. The same applies to the shared peptide fractions, which are natively used to best fit the overall signal instead of introducing quantification bias. Indeed, quantifications obtained at the finest taxonomical resolution can then be cumulated to quantify higher taxonomical levels without suffering from multiple counting of shared peptides, since the deconvolution yields quantifications cleaned from the shared signal.

The phylopeptidomic signature is based on TSMs which are MS/MS spectra assigned to taxa. This new quantitative feature in metaproteomics is the equivalent of spectral count for proteins but at the taxon level. Future improvements could be based for example on the signal derived from the intensity of the MS signal of peptides, i.e., extracted ion chromatogram (XIC), rather than MS/MS spectra. While sometimes providing (i) a larger quantification range than spectral counts and (ii) a more linear performance for large values, XIC-based quantification is sensitive to the signal complexity in the MS scans, which is a major issue in metaproteomics.

The quality of the phylopeptidomic signature is dependent on the number of available genome sequences for a given taxonomical group, and on their intrinsic quality. Thus, we recommend the inclusion of only high-quality annotated genomes in the generalist database prior to performing phylopeptidomics. Higher genome sequencing coverage of closely related species tends to substantially reduce the number of species-specific peptide sequences. This on-going trend will further degrade the performance of metaproteomic strategies relying on species-specific peptide sequences, in view of the huge worldwide resources currently devoted to sequencing organisms and improving genome coverage of extended branches of the Tree of Life. By contrast, phylopeptidomic signature-based relative quantification should render improved performances with the availability of additional genomes of higher quality, lowering the number of missing sequences that could degrade distance calculations and number of hits (PSMs/TSMs) calculations, and allowing the selection of the best genome representatives for all species.

The possible applications of the “phylopeptidomics” concept can be drawn based on possible future extensions of the method and its potential limitations. First, the database used here is the generalist NCBInr database. The methodology can be used with other databases including individual reconstructed genomes and metagenome-assembled genomes (MAGs), as soon as COGs can be predicted for phylogenetic positioning (which is possible for any full genomes). The resolution of phylopeptidomics depends on several parameters: the nature of the biological sample, the depth of the experimental dataset, and the database coverage for the taxa present in the biological sample. Here, we have shown that a relative biomass quantitation using phylopeptidomics can be obtained at species level. Sub-species and even strain-resolved levels [21] are theoretically possible if enough MS/MS spectra are recorded. The limitations in terms of maximum number of quantifiable species in the sample are an open question. If enough mass spectrometry time is allowed, mixtures of several hundred species could be analyzed by phylopeptidomics, rendering the approach consistent with fecal microbiota analysis through genome-resolved metagenomics [22]. In order to assess the limitations of the approach for strain-resolved level on the one hand, and maximum number of quantifiable organisms on the other, new experimental standards are needed. A control of the nature and exact quantities of microorganisms is required for synthetic standards representative of real microbiome samples [23]. While assembling such experimental datasets is unfortunately time- and resource-consuming, we urge the scientific community to join efforts at producing such standards for improving bioinformatic pipelines for metaproteomics in general.

## Conclusions

In conclusion, the improved evaluation of relative biomass contributions of organisms present in a microbial consortium using a phylopeptidomic signature opens new horizons for the study of microbiota and their dynamics. Assessing these biomass contributions is of considerable interest to decipher taxonomical and functional changes underlying a phenotype shift in microbiota. Because the phylopeptidomic signature applies equally well for bacteria, archaea, and eukaryotes, a large spectrum of applications is envisioned.

## Methods

### Biological material, culture conditions, and protein extraction

*S*. *bongori* CIP 82.33T (ATCC43975) and *S*. *flexneri* 2a CIP 107659 (ATCC700930) strains were obtained from the Pasteur Institute. For each strain, 40 ml of liquid tryptic soy broth medium was inoculated with a preculture grown in the same medium at an optical density of 0.2 (measured at 600 nm) and grown at 37 °C under 140 rpm agitation. Cells, 99.2 × 10^6^ for *S*. *flexneri* and 140.8 × 10^6^ for *S*. *bongori* as counted by microscopy using a Malassez cell (Rogo et Cie), were precipitated 7 h later when the cultures reached an optical density of 4.0 by adding 10 ml of 50% trichloroacetic acid (w/v) in water and centrifuging for 15 min at 2500×*g* (T40 rotor, Joun CR3i centrifuge). The cell pellet was suspended in 900 μl of 1× LDS buffer (lithium dodecyl sulfate; Invitrogen-Life Technologies) and subjected to sonication with a Vibra Cell 75042 sonicator (Bioblock Scientific) for 30 s at 40% amplitude and a total energy delivery of 1.5 kJ. As indicated in Supplementary Figure S3, for each strain and biological replicate, a volume of 10 μl of the sample (Tube 1×) was diluted in 1× LDS to obtain a 1:1 dilution (0.5×), a 1:4 dilution (0.2×), and a 1:9 dilution (0.1×). A volume of 10 μl of *S*. *flexneri* 1× extract was mixed with either 10 μl of 1× *S*. *bongori* to obtain a 1:1 mixture, 0.5× (1:0.5), 0.2× (1:0.2), or 0.1× (1:0.1). Conversely, either 10 μl of 0.5× *S*. *flexneri* extract (0.5:1), 0.2× (0.2:1), or 0.1× (0.1:1) was mixed with a volume of 10 μl of 1× *S*. *bongori* extract. In total, 33 LDS samples were prepared taking a minimum pipetted volume of 10 μl for optimal accuracy. The samples were heated at 99 °C for 5 min and were subjected to SDS-PAGE for 5 min of migration, as described [24]. The total soluble proteome of the 33 samples was reduced, treated with iodoacetamide, and in-gel digested with trypsin. The final ratios prepared in biological triplicates were 1:0, 1:0.1, 1:0.2, 1:05, 1:1, 0.5:1, 0.2:1, 0.1:1, and 0:1 in terms of relative cell counts of *S*. *flexneri* and *S*. *bongori*.

A volume of 15 μl of ZymoBIOMICS Microbial Community Standard D6300 (Zymo Research) stored in DNA/RNA Shield Zymo preservative was treated as follows: cells from the sample were harvested by centrifugation for 10 min at 10,000×*g*, diluted in 65 μl of 1× LDS, and subjected to bead-beating with a Precellys instrument (Bertin Technologies) as described [25]. After lysis, proteins were heated for 10 min at 99 °C and 25 μl of the sample was subjected to SDS-PAGE for 5 min. The whole proteome was reduced, treated with iodoacetamide, and in-gel digested with trypsin [24].

### Tandem mass spectrometry

NanoLC-MS/MS characterization of peptides from *S*. *bongori* / *S*. *flexneri* samples was performed with a LTQ-Orbitrap XL hybrid mass spectrometer (ThermoFisher) coupled to an UltiMate 3000 LC system (Dionex-LC Packings), essentially as described [26]. Peptides (10 μl) were loaded onto a reverse-phase pre-column C18 PepMap 100 column (LC Packings) and desalted online. Peptides were then resolved on a nanoscale C18 PepMap 100-capillary column (LC Packings) at a flow rate of 0.3 μl/min prior to injection into the ion trap mass spectrometer using a 90-min gradient from 4 to 40% solvent B (consisting of 0.1% HCOOH, 99.9% CH_3_CN) against solvent A, consisting of 0.1% HCOOH, 99.9% H_2_O. Full-scan mass spectra were measured from 300 to 1800 *m*/*z* in data-dependent mode using a TOP7 strategy. In this strategy, a scan cycle was initiated with a full scan of high mass accuracy in the Orbitrap analyzer followed by MS/MS scans in the linear ion trap on the seven most abundant ions. Peptides from the ZymoBIOMICS standard (2 μl) were analyzed with a Q-Exactive HF mass spectrometer (ThermoFisher) coupled to an UltiMate 3000 LC system (Dionex-LC Packings), as described [27], using a 60-min gradient of acetonitrile and a TOP20 data-dependent acquisition strategy.

### Database and MS/MS assignments

For the interpretation of MS/MS spectra, the NCBInr fasta file was downloaded (13 February 2015) as ftp://ftp.ncbi.nlm.nih.gov/blast/db/FASTA/nr.gz [28]. This version comprises 59,642,736 entries totaling 21,322,359,704 amino acids. Corresponding taxonomy files gi_taxid_prot.dmp, names.dmp, and nodes.dmp were downloaded the same day as ftp://ftp.ncbi.nlm.nih.gov/pub/taxonomy/taxdmp.zip [29]. The Mascot search engine (Matrix Science) was used for peptide inference. Molecular ion peak lists were extracted with Mascot Daemon software (version 2.5.1; Matrix Science) using the extract_msn.exe data import filter (ThermoFisher). Data import filter options were set to 400 (minimum mass), 5000 (maximum mass), 0 (grouping tolerance), 0 (intermediate scans), and 1000 (threshold), as described [26]. Peptide assignation with Mascot was done with the following parameters: full trypsin specificity, maximum of one missed cleavage, mass tolerances of 5 ppm on the parent ion and 0.5 Da on the MS/MS, static modification of carboxyamidomethylated cysteine (+ 57.0215), and oxidized methionine (+ 15.9949) as dynamic modification.

The ZymoBIOMICS sample was interpreted using a recent version (03 January 2018) of the NCBInr database, comprising 108,307,546 entries and 41,817,980,956 amino acids. The taxonomy database was also downloaded the same day. Mascot settings were the same as above except for a MS/MS mass tolerance of 0.02 Da.

### Computation of phylogenetic distances

The distance matrix was extracted from the Newick unrooted tree data [20], using Patristic distances computation in R (ape package, PatristicDistMatrix<-cophenetic(tree) command). Distances between all taxids were computed using a set of hyper-conserved COGs as described [20], with homolog sequences recovered from the set of proteins associated with each taxid using a BLAST-like approach with the DIAMOND v0.8.22.84 tool [30]. More specifically, the set of 31 COGs used was: COG0012, COG0016, COG0048, COG0049, COG0052, COG0080, COG0081, COG0087, COG0091, COG0092, COG0093, COG0094, COG0096, COG0097, COG0098, COG0099, COG0100, COG0102, COG0103, COG0172, COG0184, COG0186, COG0197, COG0200, COG0201, COG0202, COG0256, COG0495, COG0522, COG0525, and COG0533. A manually curated MSA based on the COG supervectors provided in Ciccarelli et al. [20] was used as a template to add new taxa supervectors using the following methodology. To obtain a reference MSA, the original 8090 aligned positions described in Ciccarelli et al. [20], corresponding to 191 reference organisms (23 eukaryotes, 18 archaea, and 150 bacteria), were first in-house curated to fix inter-COGs anomalies. A MUSCLE (v3.8.31) [31] alignment per COG and per superkingdom (bacteria, archaea, and eukaryota) was performed, with an additional Gblocks (v0.91b) [32] masking with the following parameters: a conserved position (including flank positions) required more than 50% of superkingdom sequences representation, the maximum number of contiguous non-conserved positions was 8, the minimum length of a block was 2 and there was no gap limitation. The final Gblocks mask used was the union of the 3 superkingdom masks. All aligned and masked COGs were then concatenated into a supervector that counted 8310 aligned positions and 180 organisms (11 redundant bacteria were removed as compared with Ciccarelli et al. [20]) to be used as a pre-aligned MSA for inter-organisms sequence similarity evaluation.

For each taxid to be added to the MSA, and for each COG to recover from its proteome, the full sequence of the closest pre-aligned taxon (in terms of number of nodes traversed in the taxonomical tree from the taxid to be added) was used as a DIAMOND query. A DIAMOND hit was validated if its *e*-value was below 0.001, identity was above 45%, and coverage was above 94%. If no hit was found fulfilling these criteria, then the reference COG sequence was used and the COG was flagged as missing. Taxa with more than 10 missing COGs were excluded from the pool of taxa with calculated distances. A total of 14,237 taxa out of 16,548 were retained. For groups of taxa with the same set of associated COGs, only one representative was retained, with other taxa in the group inheriting distance results from this taxon. COG sequences were concatenated into a supervector, which was aligned using MUSCLE in profile mode to the predefined masked MSA. Upon complete taxa addition to the alignment, CLUSTAL W (v2.1) [33], recompiled as Windows 64 b using MSYS/MinGW, was used to generate a PIM out of the MSA of all aligned taxa supervectors. PIM pair results were the distances used to evaluate the phylogenetic distance between organisms. We chose not to fix distance inconsistencies by methods such as maximum likelihood, maximum parsimony, UPGMA, or neighbor-joining after comparing signature quality using different phylogenetic tree generation and seeing degradation or marginal improvement compared with raw PIM distances, at the cost of very high time and computer power requirements (data not shown). An sqlite database was created, with distances associated with taxid pairs.

### Post-processing of Mascot dat files

Mascot dat files were parsed using the Python version of Matrix Science msparser v2.5.1 with function ms_peptidesummary [34]. Peptide-spectrum matches (PSMs) were validated with a Mascot expectation value below 0.1 using Mascot identity threshold (MIT) and allowing multiple PSMs per MS/MS spectrum.

Peptides associated with spectra were assigned to taxa using NCBI databases. In the 2015 version of NCBI data files, each header of the nr.fasta file was the concatenation of the complete set of protein identifiers matching the sequence, with a gi identifier per entry. The first gi entry per non-redundant sequence was called firstgi in our data process. The taxonomy file gi_taxid_prot.dmp was used to perform gi to taxid matching. For newly introduced “WP_” RefSeq accessions aiming at identifying each non-redundant sequence with a single multispecies accession, file ftp://ftp.ncbi.nlm.nih.gov/refseq/release/release-catalog/release68.AutonomousProtein2Genomic [35] dated 06 January 2015 was used to complement gi to taxa mapping. Corresponding Python sqlite databases created were gi2firstgi.sql and firstgi2taxid.sql, allowing fast peptide to taxid mapping through gi identifiers. For post-march 2016 versions, this database structure has been modified to match Accession.version-based NCBI files following NCBI phasing out of sequence gis.

Individual MS/MS spectra (Mascot queries) associated with peptide sequences (PSM) and protein sequences by Mascot were directly mapped to taxa using gi2firstgi and firstgi2taxid databases. In addition to the raw number of PSMs per taxon obtained, a collation of information was performed following the NCBI taxonomical tree from direct assignation to “canonical” taxonomical levels: species, genus, family, order, class, phylum, and superkingdom. For each taxon at each level, the total peptide sequences and total PSMs were counted, as well as specific or unique peptide sequences and corresponding specific PSMs.

For the 2018 version used to process the ZymoBIOMICS sample, accessions were used as sequence identifiers, and the mapping of accessions to taxa was performed using assembly_summary_refseq.txt and assembly_summary_genbank.txt files downloaded from ftp://ftp.ncbi.nlm.nih.gov/genomes/ASSEMBLY_REPORTS [36], to map taxids to RefSeq assemblies (GCF) and GenBank assemblies (GCA), then the GCF/GCA *_assembly_report.txt files to map GCF/GCA to nucleotides, and the *_genomic.gff.gz files to map GCF/GCA to protein accessions.

### Phylopeptidomics signature fit

Taxa were filtered retaining those with MS/MS attribution, number of missing COGs lower than 10, and an assembly level quality “Complete genome” as extracted from file assembly_summary_refseq.txt ([36]). The matrix of taxa pair distances and the vector of the number of PSMs per taxon were used to calculate a phylopeptidomic signature fit. The two distance vectors for *S*. *flexneri 2a str*. *301* and *S*. *Bongori NCTC 12419* were extracted from the full matrix, and the corresponding *x* values were used to compute the sum of the two theoretical signatures (function *y* = *N*×[*A* × exp(−*x*/*a*) + (1 − *A*) × exp(− *x*/*b*)]) compared with actual MS/MS taxa data points. The “L-BFGS-B” method from the Python package scipy.optimize was used to best fit the sum of signature functions *y* = *N*×[*A* × exp(− *x*/*a*) + (1 − *A*) × exp(− *x*/*b*)] to the proteomic signal. Default values for the variables were *A* = 0.45, *a* = 0.013, *b* = 0.082, and *N* = #TSMs (taxon). Normalization was applied to the parameters during search space exploration, using the correction factors 1/*A*, 1/*a*, 1/*b*, and 1/100. Bounds used were: default value ± 0.05 for *A*, ± 0.001 for *a* and *b*, and 0 to #TSMs × 5 for *N*. The objective function was the sum of quadratic errors between taxa data points and the corresponding estimate based on the sum of signatures. The tolerance for termination was set to 0.01. A similar setup was used to obtain the fit of the ZymoBIOMICS Microbial Community Standard D6300. Organisms to be quantified were set as *Pseudomonas aeruginosa*, *Escherichia coli*, *Salmonella enterica*, *Lactobacillus fermentum*, *Enterococcus faecalis*, *Staphylococcus aureus*, *Listeria monocytogenes*, *Bacillus subtilis*, *Saccharomyces cerevisiae*, and *Cryptococcus neoformans*.

## Supplementary information

**Additional file 1: Supplementary Figure S1.** Phylogenetic distances computed using COGs compared to 16S based RDP distances.

**Additional file 2: Supplementary Figure S2.** The linear combination of signatures fits almost perfectly the overall proteomic signal acquired on the ZymoBIOMICS Microbial Community Standard.

**Additional file 3: Supplementary Figure S3.** Protocol design for accurate *Shigella / Salmonella* ratios.

**Additional file 4: Supplementary Table SI.** Proteomic results for the 27 *S. flexneri/S*. *bongori* samples.

**Additional file 5: Supplementary Table SII.** Relative quantification of *S*. *flexneri* and *S*. ***bongori*** results for the 27 *S. flexneri*/*S*. *bongori* samples.

**Additional file 6: Supplementary Table SIII.** Proteins identified and SC quantified in the 27 *S*. *flexneri/S. bongori* samples.

**Additional file 7: Supplementary Table SIV.** Quantitation of a mock community sample with same protein content per organism from Kleiner et al.

## Data Availability

The mass spectrometry proteomics data have been deposited at the ProteomeXchange Consortium via the PRIDE partner repository with the dataset identifier PXD015500.

## Abbreviations

COGs: Clusters of orthologous groups of proteins
GCA: GenBank assemblies
GCF: RefSeq assemblies
LDS: Lithium Dodecyl Sulfate
MIT: Mascot identity threshold
MSA: Multiple sequence alignment
NCBInr: National Center for Biotechnology Information non-redundant
PIM: Percent identity matrix
PSMs: Peptide-to-spectrum matches
TSMs: Taxon-to-spectrum matches
