# Supplementary figures and images for: Estimating relative biomasses of organisms in microbiota using “phylopeptidomics”
Source: Microbiome. 2020 Mar 6;8:30. doi: 10.1186/s40168-020-00797-x (PMC7060547; doi:10.1186/s40168-020-00797-x)

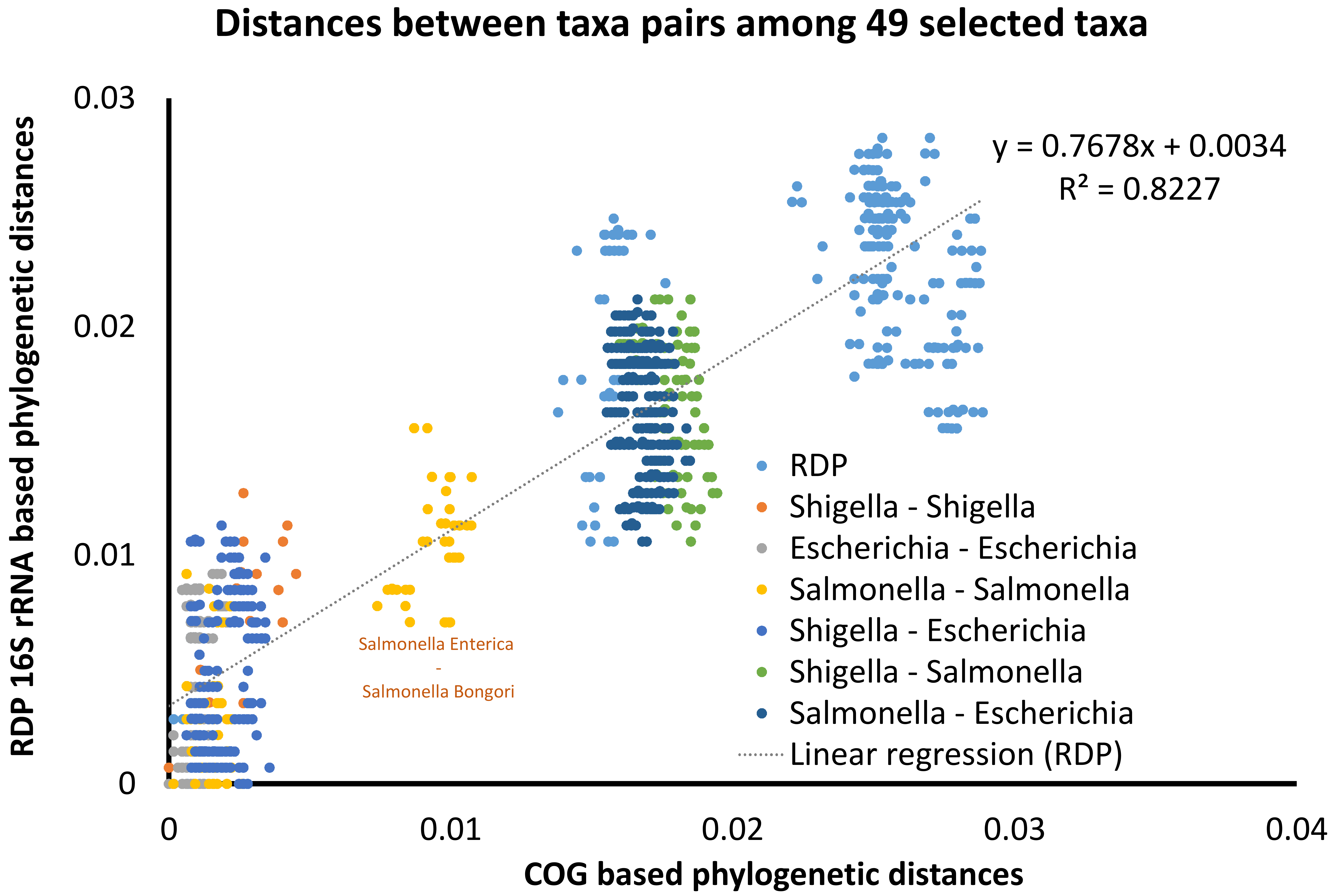

Supplement: Supplementary file 2 — Additional file 1: Supplementary Figure S1. Phylogenetic distances computed using COGs compared to 16S based RDP distances. [file 40168_2020_797_MOESM1_ESM.tif]

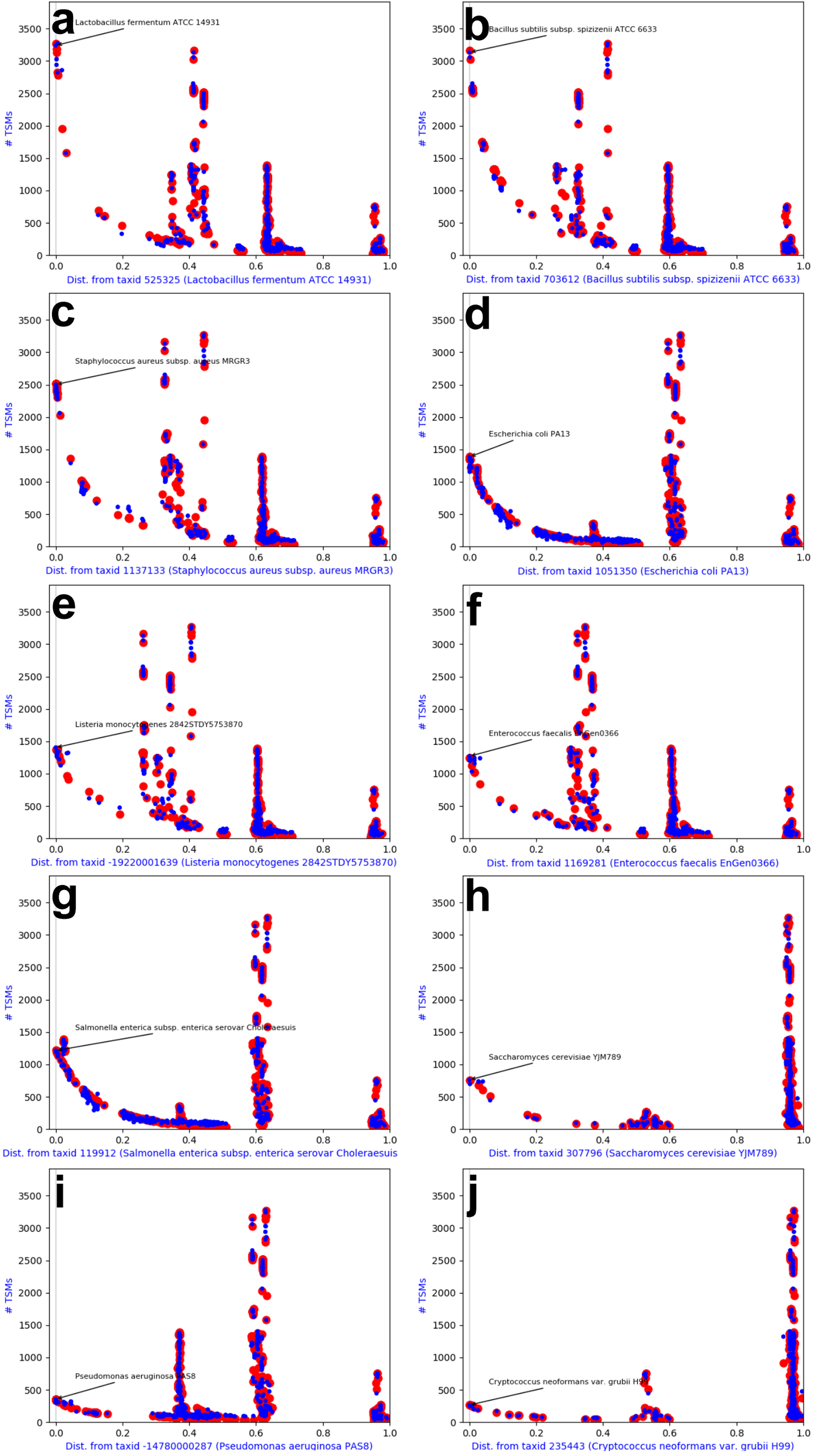

Supplement: Supplementary file 3 — Additional file 2: Supplementary Figure S2. The linear combination of signatures fits almost perfectly the overall proteomic signal acquired on the ZymoBIOMICS Microbial Community Standard. [file 40168_2020_797_MOESM2_ESM.tif]

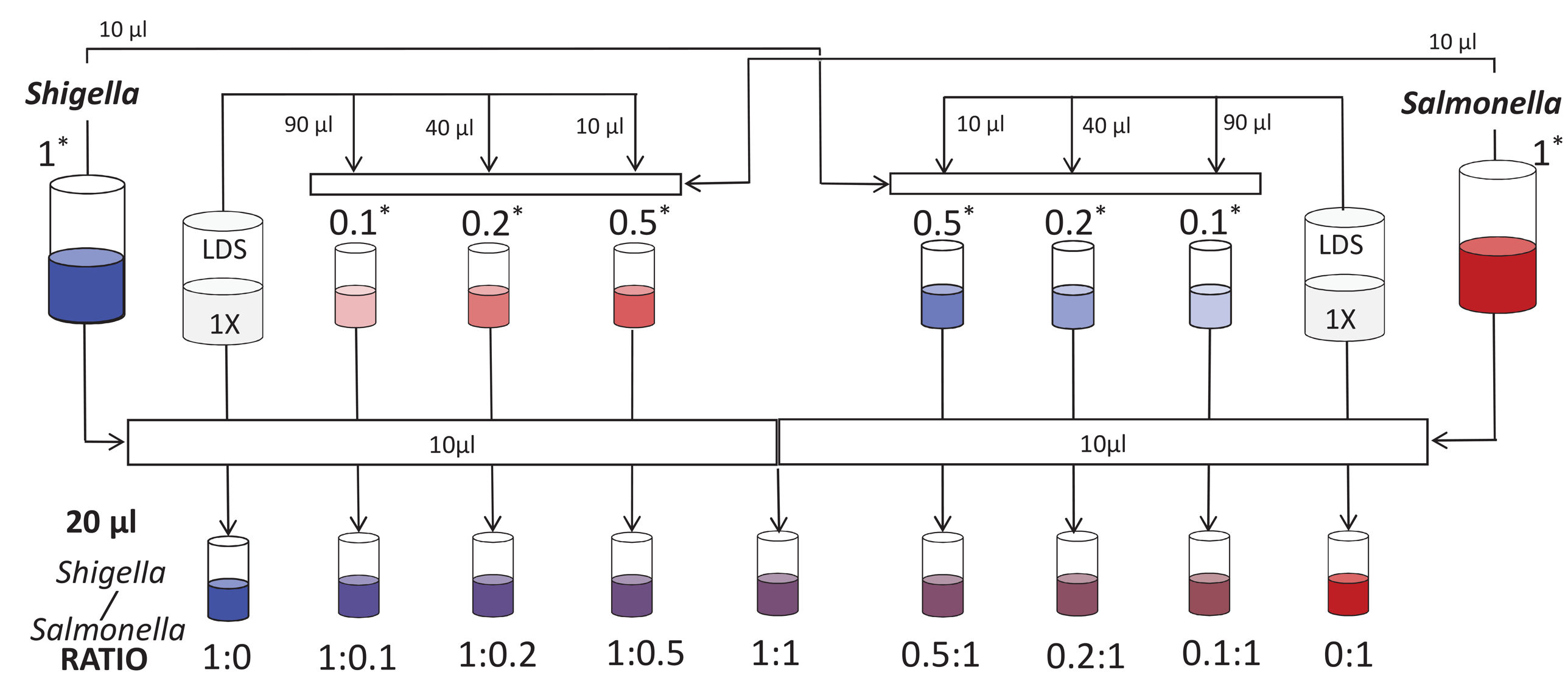

Supplement: Supplementary file 4 — Additional file 3: Supplementary Figure S3. Protocol design for accurate Shigella / Salmonella ratios. [file 40168_2020_797_MOESM3_ESM.tif]
